# Reversal of type 1 diabetes via islet β cell regeneration following immune modulation by cord blood-derived multipotent stem cells

**DOI:** 10.1186/1741-7015-10-3

**Published:** 2012-01-10

**Authors:** Yong Zhao, Zhaoshun Jiang, Tingbao Zhao, Mingliang Ye, Chengjin Hu, Zhaohui Yin, Heng Li, Ye Zhang, Yalin Diao, Yunxiang Li, Yingjian Chen, Xiaoming Sun, Mary Beth Fisk, Randal Skidgel, Mark Holterman, Bellur Prabhakar, Theodore Mazzone

**Affiliations:** 1 Section of Endocrinology, Diabetes & Metabolism, Department of Medicine, University of Illinois at Chicago, 1819 West Polk Street, Chicago, IL 60612 USA; 2 Section of Endocrinology, General Hospital of Jinan Military Command, 25 Shifan Road, Jinan, Shandong 250031, P.R. China; 3 Stem Cell Treatment Center, General Hospital of Jinan Military Command, 25 Shifan Road, Jinan, Shandong 250031, P.R. China; 4 Section of Blood Transfusion, General Hospital of Jinan Military Command, 25 Shifan Road, Jinan, Shandong 250031, P.R. China; 5 Section of Molecular Diagnostics, General Hospital of Jinan Military Command, 25 Shifan Road, Jinan, Shandong 250031, P.R. China; 6 Section of Neuronology, Jinan Central Hospital, 105 Jiefang Road, Jinan, Shandong 250020, P.R. China; 7 Jinan Tianhe Stem Cell Biotechology Co. Ltd., 750 Shunhua Road, Jinan, Shandong 250055, P.R. China; 8 Texas Cord Blood Bank, 6211 IH-10 West, San Antonio, TX 78201 USA; 9 Department of Pharmacology, University of Illinois at Chicago, 835 South Wolcott Avenue, Chicago, IL 60612 USA; 10 Department of Surgery, University of Illinois at Chicago, 420 NE GLen Oak, Suite 201, Peoria, IL 61603 USA; 11 Department of Immunology and Microbiology, University of Illinois at Chicago, 835 South Wolcott Avenue, Chicago, IL 60612 USA

## Abstract

**Background:**

Inability to control autoimmunity is the primary barrier to developing a cure for type 1 diabetes (T1D). Evidence that human cord blood-derived multipotent stem cells (CB-SCs) can control autoimmune responses by altering regulatory T cells (Tregs) and human islet β cell-specific T cell clones offers promise for a new approach to overcome the autoimmunity underlying T1D.

**Methods:**

We developed a procedure for Stem Cell Educator therapy in which a patient's blood is circulated through a closed-loop system that separates lymphocytes from the whole blood and briefly co-cultures them with adherent CB-SCs before returning them to the patient's circulation. In an open-label, phase1/phase 2 study, patients (n = 15) with T1D received one treatment with the Stem Cell Educator. Median age was 29 years (range: 15 to 41), and median diabetic history was 8 years (range: 1 to 21).

**Results:**

Stem Cell Educator therapy was well tolerated in all participants with minimal pain from two venipunctures and no adverse events. Stem Cell Educator therapy can markedly improve C-peptide levels, reduce the median glycated hemoglobin A_1_C (HbA_1_C) values, and decrease the median daily dose of insulin in patients with some residual β cell function (n = 6) and patients with no residual pancreatic islet β cell function (n = 6). Treatment also produced an increase in basal and glucose-stimulated C-peptide levels through 40 weeks. However, participants in the Control Group (n = 3) did not exhibit significant change at any follow-up. Individuals who received Stem Cell Educator therapy exhibited increased expression of co-stimulating molecules (specifically, CD28 and ICOS), increases in the number of CD4^+^CD25^+^Foxp3^+ ^Tregs, and restoration of Th1/Th2/Th3 cytokine balance.

**Conclusions:**

Stem Cell Educator therapy is safe, and in individuals with moderate or severe T1D, a single treatment produces lasting improvement in metabolic control. Initial results indicate Stem Cell Educator therapy reverses autoimmunity and promotes regeneration of islet β cells. Successful immune modulation by CB-SCs and the resulting clinical improvement in patient status may have important implications for other autoimmune and inflammation-related diseases without the safety and ethical concerns associated with conventional stem cell-based approaches.

**Trial registration:**

ClinicalTrials.gov number, NCT01350219.

## Background

In Type 1 diabetes (T1D), autoimmune destruction of pancreatic islet β cells reduces an individual's ability to regulate blood glucose, ultimately resulting in poor blood circulation, heart disease, stroke, infection, kidney failure, and often premature death. Each day, millions of patients with T1D receive insulin injections to survive, but these injections do nothing to address the underlying T cell-mediated autoimmune dysfunction. For the past 25 years, attempts to address the underlying autoimmunity have been unsuccessful [1] due to the polyclonal nature of the autoimmune response and the global challenges of immune regulation in T1D patients [1-5]. Combinations of individual approaches have been proposed to address these challenges [2,6-8], but adherence to these approaches will be complicated and costly. Alternative approaches are needed. Stem cells have been touted as a means of replacing lost pancreatic islet β cells and curing T1D, but this approach is doomed in the absence of a treatment for the underlying autoimmune response.

While traditional stem cell therapy is not likely to be effective for long-term treatment of T1D, recent studies suggest that alternative approaches using stem cells may overcome the autoimmune component of the disease. Human cord blood-derived stem cells (CB-SCs) and mesenchymal stem cells have been shown to modulate immune activity *in vitro *[9-13]. Subsequent studies have demonstrated that CB-SCs can be used to alter immune function and improve markers of T1D in nonobese diabetic mice (NOD) [14], and CB-SCs have been shown to modulate the immune function of T1D patient-derived islet β cell-specific pathogenic T cell clones in co-culture [9]. Studies in animal models also suggest that CB-SC treatment may allow the patient to regenerate the native population of islet β cells without stem cell transplantation [9,14,15]. To translate these findings into a clinically feasible therapy, we developed a novel process to re-educate a patient's lymphocytes through co-culture with CB-SCs. If shown to be safe and effective, immune modulation by CB-SCs has the potential to address T1D and other autoimmune diseases while reducing risk to the donor, minimizing ethical concerns, and avoiding graft-versus-host disease [9].

## Methods

### Patients

T1D subjects receiving care through the Section of Endocrinology at the General Hospital of Jinan Military Command (Jinan, Shandong, China) were enrolled in a phase 1/phase 2, open-label clinical trial conducted from October 2010 through January 2011. With oversight from a planning committee, the principal investigator designed the trial and received ethical approval for the clinical treatment protocol and consent form from the General Hospital of Jinan Military Command (Jinan, Shandong, China) and ethical approval for the *in vitro *study protocol and consent form from the University of Illinois at Chicago Institutional Review Board. Written informed consent was obtained from each participant. The trial was conducted with 15 subjects with established T1D (mean duration: 8.5 ± 6.4 years). Patients were qualified for enrollment if they met the 2010 diagnosis standards of the American Diabetes Association and a blood test confirmed the presence of at least one autoantibody to pancreatic islet β cells. Exclusion criteria included clinically significant liver, kidney, or heart disease; pregnancy; immunosuppressive medication; viral diseases; or diseases associated with immunodeficiency.

### Stem Cell Educator design

**Figure 1:**
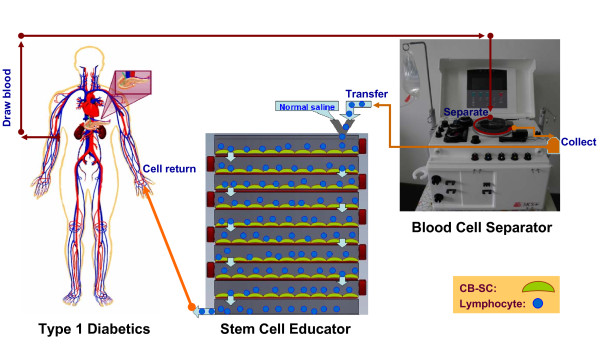
**Overview of Stem Cell Educator therapy**. A T1D participant (left) is connected to a Blood Cell Separator (right) and the Stem Cell Educator (bottom center) to form a closed system. Lymphocytes isolated from the T1D participant by the Blood Cell Separator travel through the Stem Cell Educator where they come in contact with CB-SCs attached to the interior surfaces of the device. Educated lymphocytes are returned to the patient's blood circulation. CB-SCs, cord blood stem cells; T1D, type 1 diabetes.

In previous studies we isolated multipotent cord blood stem cells (CB-SCs) from human cord blood [16]. The CB-SCs display embryonic cell markers (for example, transcription factors OCT-4 and Nanog, stage-specific embryonic antigen (SSEA)-3 and SSEA-4) and leukocyte common antigen CD45, but they are negative for blood cell lineage markers [9,16]. We identified a hydrophobic material from FDA-approved (USP Class VI) Petri dishes that tightly binds CB-SCs without interfering with their immune modulating capability. We designed a chamber for co-culture of lymphocytes and CB-SCs that includes nine discs of the material with adherent CB-SCs sandwiched between a top cover plate and a bottom collecting plate (Figure 1). The device was manufactured in a Class 100 K clean room and gamma-irradiated prior to introducing CB-SCs [16]. In the Stem Cell Educator, lymphocytes separated from a patient's blood are slowly passed through the stacked discs of material with adherent CB-SCs and lymphocytes collected through a hole in the bottom plate are returned to the patient. The materials used to produce the device are approved for *in vivo *use according to the United States Pharmacopeia (that is, Grade Class VI Plastic).

### CB-SC culture

Human cord blood units derived from healthy donors were purchased from the Maternal and Child Health Hospital (Jinan, Shandong, China). All cord blood samples were screened for alanine aminotransferase and pathogenic antigen antibodies (including anti-HCV, anti-HBsAg, anti-HIV, and anti-syphilis Abs), and only pathogen-free cord blood units were used for isolating CB-SCs. Human cord blood-derived stem cells (CB-SC) were generated as previously described with the following modifications [14,16]. Cord blood mononuclear cells were plated in serum-free culture medium (Lonza, Walkersville, MD) and incubated at 37°C, in 8% CO_2_. After 2 to 3 weeks, CB-SCs growing at 80% to 90% confluence were prepared for clinical trial. Endotoxin level was < 0.05 EU/ml.

### Treatment and follow-up

Twelve participants received a single treatment with the Stem Cell Educator (Tianhe Stem Cell Biotechnology^®^, Jinan, China), and three received a single treatment with the Stem Cell Educator without adherent CB-SCs (that is, sham or process-only control) (Figure 1). A 16-gauge IV needle was placed in the left (or right) median cubital vein, and the patient's blood was passed through a Blood Cell Separator MCS+ (Haemonetics^®^, Braintree, MA, US) at 35 mL/min for 6 to 7 hours to isolate lymphocytes in accordance with the manufacturer's recommended protocol. The collected lymphocytes were transferred into the device for exposure to allogeneic CB-SCs (or process control without CB-SCs), and other blood components were returned to the patient. After 2 to 3 hours in the device, lymphocytes were returned to the patient's circulation via a dorsal vein in the hand under gravity flow control (2 to 3 mL/min) with physiological saline. Approximately 10,000 mL of blood was processed during the procedure resulting in approximately two repeated educations for the lymphocyte fraction. Patients were hospitalized for two days to monitor temperature and conduct routine laboratory blood tests for adverse reactions following treatment. Follow-up visits were scheduled 4, 12, 24, and 40 weeks after treatment for clinical assessments and laboratory tests (Additional file 1).

### Study end points

The primary study end points were: 1) feasibility of the Stem Cell Educator therapy; 2) safety of the therapy through 12 weeks post treatment; and 3) preliminary evaluation of the efficacy of the therapy for improving β cell function through 24 weeks. Pancreatic islet β cell function was assessed by measuring basal and glucose-stimulated C-peptide production over time, as described elsewhere [17,18]. Metabolic control was monitored throughout the study. The secondary study end point was evidence of the efficacy of the therapy in modulating autoimmunity. Baseline blood samples were collected prior to Stem Cell Educator therapy. Detailed descriptions of the methods are included in the Supplementary Appendix.

### Statistics

An intention-to treat approach was used, with 12 of 15 patients undergoing Stem Cell Educator therapy and the remaining 3 patients undergoing sham therapy without CB-SCs in the Educator. All patients were included in the safety analyses. The primary efficacy end point was the change in C-peptide secretion between baseline and follow-up.

## Results

### Feasibility and safety of Stem Cell Educator therapy

Fifteen T1D patients were enrolled (baseline characteristics presented in Table 1). Median age was 29 years (range: 15 to 41), and median diabetic history was 8 years (range: 1 to 21). Participants were randomly assigned to receive Stem Cell Educator therapy (n = 12) or sham therapy (n = 3). Each participant received one treatment. Based on fasting C-peptide levels (a by-product of insulin biosynthesis, as an indicator for islet β cell function), participants in the treatment group were characterized as having moderate T1D with some residual β cell function (n = 6, Group A) or severe T1D with no residual pancreatic islet β cell function (n = 6, Group B) (Table 1). All Control Group participants had moderate T1D.

**Table 1:** Characteristics of the T1D subjects before treatment

Patient **No**.	Age	Gender	Marriage	History (year)	Height (cm)	Body weight (kg)	C-peptide (ng/ml)*	Auto-Antibodies				HbA1C (%)	Insulin dose (U/day)
								IA-2A	GAD	ICA	IAA		
**Group A: Long-standing patients having some residual islet β cell function and received therapy with CB-SC**													
1	17	F	No	5	145	35	0.30	-	+	-	-	12.3	52
2	23	F	No	5	167	59	0.56	-	+	-	-	6.6	50
3	38	F	Yes	11	160	60	0.12	-	+	-	-	7.3	23
4	39	F	Yes	1	156	49	0.636	+	+	+	-	11.3	22
5	31	M	Yes	14	170	70	0.18	+	+	-	-	6.5	30
6	30	M	Yes	2	182	70	0.18	-	+	-	-	8.4	40
**Mean** **(SD)**	**30** **(9)**			**6** **(5)**	**163** **(12.7)**	**57** **(13.4)**	**0.33** **(0.22)**					**8.7** **(2.5)**	**36.2** **(13.2)**
**Group B: Long-standing severe patients with no residual islet b cell function and received therapy with CB-SC**													
7	40	M	Yes	17	170	72.5	0.01	-	+	-	-	8.8	48
8	15	F	No	5	163	65	0.01	-	+	-	-	15.5	50
9	21	F	No	4	168	65	0.01	+	-	-	-	9.9	46
10	23	F	No	12	162	80	0.01	+	+	+	-	16.5	60
11	40	F	Yes	21	160	67	0.01	+	-	-	-	8.6	37
12	21	F	No	5	157	56	0.01	-	+	-	-	13.6	50
**Mean** **(SD)**	**27** **(11)**			**11** **(7)**	**163** **(4.9)**	**67.6** **(8.1)**	**0.01** **(0)**					**12.2** **(3.5)**	**48.5** **(7.4)**
**Control group: Long-standing patients having some residual islet β cell function and received sham therapy**													
13	35	M	Yes	1	178	73.5	0.37	+	-	+	-	9.8	30
14	41	M	Yes	14	165	65	0.55	-	+	-	-	6.4	48
15	24	M	No	3	175	58	0.3	-	+	-	-	10.7	48
**Mean** **(SD)**	**33** **(9)**			**6** **(7)**	**173** **(6.8)**	**65.5** **(7.8)**	**0.41** **(0.13)**					**9.0** **(2.3)**	**42** **(10.4)**

*The level of 0.01 ng/ml is the minimum detectable level (sensitivity) of C-peptide by radioimmunoassay (RIA). To convert C-peptide value to nmol/L, multiply the ng/ml by 0.331.

No participants experienced any significant adverse events during the course of treatment. Most patients experienced mild discomfort during venipuncture and some soreness of the arm during apheresis, but discomfort and soreness resolved quickly following the conclusion of the procedure. Twenty-four hours post treatment, no significant difference was noted in white blood cell counts relative to baseline (total white blood cell count: 6.95 × 10^9^/L ± 1.98 versus 6.39 × 10^9^/L ± 1.72, *P = *0.38; granulocytes: 3.79 × 10^9^/L ± 1.43 versus 3.66 × 10^9^/L ± 1.05, *P = *0.77; lymphocytes: 2.31 × 10^9^/L ± 0.9 versus 2.08 × 10^9^/L ± 0.67, *P = *0.40; monocytes: 0.49 × 10^9^/L ± 0.13 versus 0.46 × 10^9^/L ± 0.10, *P = *0.48). Participants' body temperatures were not significantly changed during the two-day post-treatment observation (36.44°C ± 0.24 versus 36.5°C ± 0.22, n = 15, *P = *0.35). No changes were observed in blood cell count or temperature at the 12-week follow-up.

CB-SCs are tightly adherent [9,16] and not expected to escape the device. To confirm CB-SCs are completely retained in the Educator and not transferred to the patient, we examined cells leaving the device to check for SSEA-3, a CB-SC-specific marker. Flow cytometry confirmed the absence of SSEA-3 in cells leaving the Educator (Additional file 1: Figure S1). These data indicate that the cells returned to the patients are autologous. Additionally, HLA matching is not required prior to Stem Cell Educator therapy because CB-SCs are not transferred to the patient and because CB-SCs have very low immunogenicity [9,13,16]. Thus, Stem Cell Educator therapy is a very safe approach.

### Efficacy outcomes in improving beta cell function

**Figure 2:**
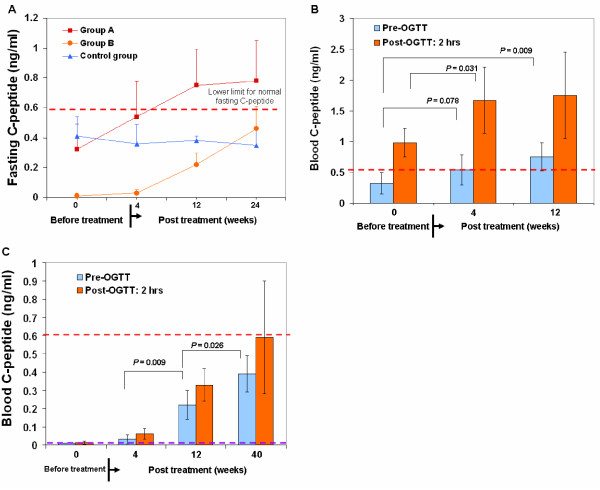
**Improvement of β-cell function by Stem Cell Educator therapy**. (**A**) Fasting C-peptide levels of T1D participants over 24 weeks. Group A and Group B participants (n = 6 per group) received one Stem Cell Educator treatment. Control group participants (n = 3) received sham therapy (no CB-SCs in the Stem Cell Educator). (**B**) 12-week follow-up C-peptide levels after OGTT at 2 hours in Group A T1D subjects with some residual β cells. (**C**) Comparison of C-peptide levels at glucose challenge after 40-week follow-up in Group B T1D subjects. The dashed red line indicates the lower limit for normal C-peptide levels in Chinese populations. The dashed purple line indicates the minimum detectable level (sensitivity) of C-peptide by radioimmunoassay (RIA). CB-SCs, cord blood stem cells; OGTT, oral glucose tolerance test; T1D, type 1 diabetes.

Participants in Group A (that is, those with moderate T1D and some residual β cell function) exhibited improved fasting C-peptide levels at 12 and 24 weeks post-treatment (Figure 2A and 2B, Table 2), and participants in Group B (that is, those with severe T1D and no residual pancreatic islet β cell function) exhibited successive improvement in fasting C-peptide levels at each follow-up (Figure 2A and 2C, Table 2). C-peptide response following a 75-g oral glucose tolerance test (OGTT) improved among Group A participants at 4 and 12 weeks (Figure 2B). Notably, participants in Group B exhibited essentially no C-peptide production following glucose challenge at baseline (that is, less than the minimum sensitivity of 0.01 ng/ml at all time points) but demonstrated marked improvement at 12 weeks (Figure 2C, Table 2). Improvement was maintained through the final follow-up (that is, 40 weeks post-treatment; *P = *0.026) (Figure 2C). Participants in the Control Group did not exhibit significant change at any follow-up (Figure 2A, Table 2).

**Table 2:** Changes in C-peptide levels of the T1D subjects after treatment at 12 weeks.

**Patient No**.	C-peptide (ng/ml)	Changes in C-peptide levels (ng/ml)
**Group A:**		

1	0.56	↑ 0.26

2	0.98	↑ 0.42

3	0.54	↑ 0.42

4	1.1	↑ 0.46

5	0.52	↑ 0.34

6	0.78	↑ 0.60

**Mean**	**0.75**	**↑ 0.42**

**(SD)**	**(0.24)**	**(0.12)**

**Group B**		

7	0.25	↑ 0.24

8	0.11	↑ 0.10

9	0.28	↑ 0.27

10	0.43	↑ 0.42

11	0.11	↑ 0.10

12	0.12	↑ 0.11

**Mean**	**0.22**	**↑ 0.21**

**(SD)**	**(0.13)**	**(0.13)**

**Control Group**		

13	0.41	↑ 0.04

14	0.38	↓ 0.17

15	0.35	↑ 0.05

**Mean**	**0.38**	**↓ 0.03**

**(SD)**	**(0.03)**	**(0.12)**

Levels of plasma C-peptide before treatment served as baseline for comparison. The level of C-peptide was increased (↑); the level of C-peptide was decreased (↓).

Consistent with improved β cell function, the median daily dose of insulin was reduced 38% at 12 weeks post-treatment in Group A (36 ± 13.2 units/day at baseline versus 22 ± 1.8 units/day 12 weeks post-treatment) and 25% in Group B (48 ± 7.4 units/day at baseline versus 36 ± 4.4 units/day 12 weeks post- treatment), but no change was observed in the Control Group. The reduced daily dose of insulin in Group A and B was maintained through the last follow-up for this measure (24 weeks). The median glycated hemoglobin (HbA_1_C) in Group A was significantly lowered from 8.73% ± 2.49 at baseline to 7.67% ± 1.03 at 4 weeks post-treatment (*P = *0.036) and to 6.82% ± 0.49 at 12 weeks post-treatment (*P = *0.019). The median HbA_1_C in Group B was reduced 1.68% ± 0.42 at 12 weeks post-treatment, but no change was observed in the Control Group (9.0% ± 2.3 at baseline versus 8.7% ± 1.9 at 12 weeks post-treatment, *P = *0.86). Thus, the *ex vivo *immune education of CB-SC leads to regeneration of islet β cells and improvement of β cell function in long-standing T1D subjects.

### Efficacy outcomes in autoimmune control

**Figure 3:**
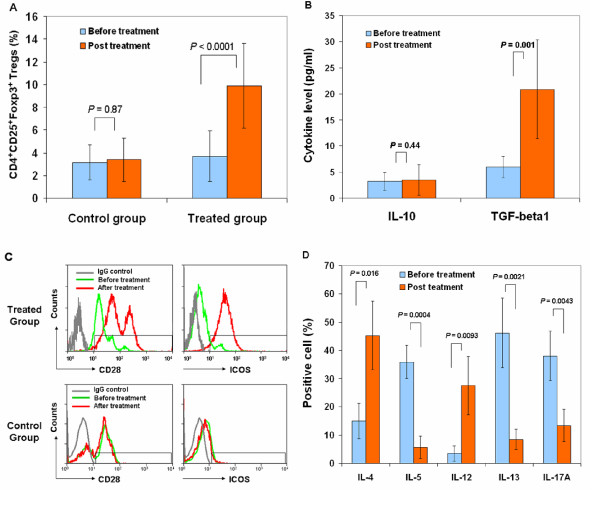
**Markers of immune function in T1D patients after Stem Cell Educator therapy**. Patient lymphocytes were isolated from peripheral blood by Ficoll-Hypaque (γ = 1.077) for flow cytometry analyses in T1D patients at baseline and 4 weeks after Stem Cell Educator therapy. Isotype-matched IgG served as control. **(A) **Flow Analysis of CD4^+^CD25^+^Foxp3^+ ^Tregs demonstrating an increase in the percentage of Tregs at 4 weeks post-treatment. **(B) **Cytokine ELISAs demonstrating an increase in TGF-β1 but not IL-10 at 4 weeks post treatment. **(C) **Flow cytometry on co-stimulating molecules indicating increases in CD28 and ICOS at 4 weeks post-treatment with Stem Cell Educator therapy (top panels). Control group failed to show increases (bottom panels). **(D) **Flow analysis of intra-cellular cytokines demonstrating differential effects on key interleukins at 4 weeks post-treatment. Data are representative of preparations from all T1D participants (n = 12) that received Stem Cell Educator therapy. ELISA, enzyme-linked immunosorbent assay; ICOS, inducible costimulator; IgG, immunoglobulin G; IL10, interleukin 10; T1D, type 1 diabetes; Tregs, regulatory T cells.

Next, we explored mechanisms underlying CB-SC-mediated immune modulation. Regulatory T lymphocytes (Tregs) play a crucial role in maintaining homeostasis and self-tolerance by inhibiting the action of autoreactive effector T cells [14,19,20], but previous attempts to manipulate Tregs for clinical applications have been problematic [21]. We measured changes in the percentage of CD4^+^CD25^+^Foxp3^+ ^Tregs in peripheral blood of participants following Stem Cell Educator treatment. The percentage of Tregs in peripheral blood of participants was significantly increased 4 weeks after Stem Cell Educator therapy (Figure 3A), whereas the percentage of Tregs in peripheral blood of participants receiving sham therapy was unchanged from baseline (Figure 3A). TGF-β1 has also been implicated in Treg-mediated immune suppression [22] as well as in maintenance of self-tolerance in T1D animal models subjected to stem cell-mediated immune modulation [9,15,23]. We examined TGF-β1 and IL-10 expression to explore whether these pathways are activated following Stem Cell Educator therapy. Participants in the treatment group exhibited significant increases in plasma level of TGF-β1 at the 4-week follow-up (*P *= 0.001, Figure 3B) but did not exhibit changes in the plasma level of IL-10 (*P *= 0.44, Figure 3B). Both TGF-β1 and IL-10 failed to show changes in the control group.

We also examined levels of CD28 [24-28] and inducible costimulator (ICOS) [29,30], which are essential for the establishment, maintenance and efficacy of Tregs [24-32]. Flow cytometry revealed an increase in CD28 and ICOS in lymphocytes 4 weeks after Stem Cell Educator therapy (Figure 3C), but levels of both molecules were unchanged in participants receiving sham therapy (Figure 3C). We also noted other changes at the 4-week follow-up consistent with improved helper T cell 1 (Th1) and Th2-mediated immune function (Figure 3D). Expression of IL-4 and IL-12 was significantly increased (*P *= 0.016 and *P *= 0.0093, respectively) and expression of IL-5 and IL-13 was decreased (*P *= 0.00039 and *P *= 0.00206, respectively). The production of pro-inflammatory IL-17A was also decreased 4 weeks after treatment (Figure 3D, P = 0.0043). No changes were observed in levels of these cytokines in participants who received sham therapy (Additional file 1: Figure S2).

**Figure 4:**
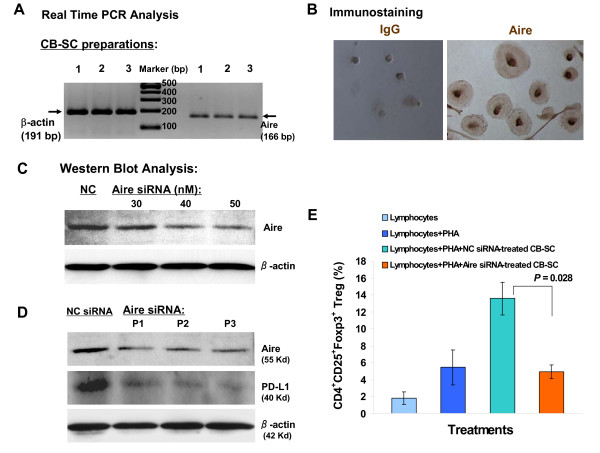
**Characterization of Aire in CB-SC**. (**A**) Expression of Aire mRNA in CB-SCs. Real time PCR analysis for Aire mRNA expression followed by electrophoresis in 2% agarose gel. Data are representative of three CB-SC preparations. (**B**) Immunocytochemistry for Aire. Isotype-matched IgG served as control (left) for Aire staining (right) with magnification ×200. (**C**) Western blot shows the dose-dependent knockdown response of Aire following siRNA treatment. **(D) **Effects of Aire knockdown on PD-L1. Western blot demonstrates decreased expression of program death ligand-1 (PD-L1) in CB-SC following knockdown of Aire expression by siRNA. CB-SC cells transfected with negative control siRNA (NC siRNA) served as control for three pairs of human Aire-specific siRNA (P1, P2, and P3) at optimal concentration (50 nM). Representative data of those obtained from five experiments. **(E) **Effects of Aire knockdown on co-cultured lymphocytes. Flow analysis of Treg population following culture of lymphocytes alone, in the presence of phytohaemagglutinin (PHA, 10 μg/ml), in the presence of PHA and NC siRNA-treated CB-SCs, and in the presence of PHA and Aire siRNA (50 nM)-treated CB-SCs. Representative data obtained from three experiments. Aire, autoimmune regulator; CB-SCs, cord blood stem cells; IgG, immunoglobulin G: PCR, polymerase chain reaction; PHA, phytohaemagglutinin; siRNA, small interfering RNA; T1D, type 1 diabetes; Tregs, regulatory T cells.

Autoimmune regulator (Aire), usually expressed in thymic medullary epithelial cells, plays an important role in immune tolerance by mediating ectopic expression of peripheral self-antigens and mediating the deletion of auto-reactive T cells [33,34]. We found that CB-SCs express Aire (Figures 4A and 4B). To determine the Aire function in CB-SC, we used three pairs of human Aire-specific small interfering RNAs (siRNA) to knock down Aire expression in CB-SCs. Western blots confirmed knockdown of Aire protein expression (Figure 4C) and a corresponding reduction in the expression of programmed death ligand-1 (PD-L1) that contributes to the immune modulation of CB-SC [13,35] (Figure 4D). Knockdown of Aire also reduced the percentage of Tregs in the co-cultured lymphocyte population (*P = *0.028) (Figure 4E). The data indicate that Aire is involved in immune modulation and induction of immune tolerance following Stem Cell Educator therapy.

## Discussion

The present studies demonstrate the safety and therapeutic efficacy of Stem Cell Educator therapy in T1D patients. The device, essentially a stack of specially-designed Petri dishes with adherent CB-SCs, functions as part of a closed-loop system that circulates a patient's blood through a blood cell separator, briefly co-cultures the patient's lymphocytes with CB-SCs *in vitro*, and returns the educated lymphocytes to the patient's circulation. Through secreted and cell-surface signaling molecules, the CB-SCs educate the lymphocytes passing through the device [9]. The cells returned to the patients are autologous lymphocytes that have been treated (or educated) by CB-SCs. The Stem Cell Educator therapy requires only two venipunctures, carries a lower risk of infection than a typical blood transfusion, and does not introduce stem cells or reagents into patients. In addition, CB-SCs have very low immunogenicity, eliminating the need for human leukocyte antigen (HLA) matching prior to treatment [9,13,16]. Thus, this innovative approach may provide CB-SC-mediated immune modulation therapy for multiple autoimmune diseases while mitigating the safety and ethical concerns associated with other approaches [4,11,21,36]. The relative simplicity of the approach may also provide cost and time savings relative to other approaches.

Results from this trial confirm prior studies indicating that the adherence of CB-SCs could be exploited to develop therapies that do not introduce the CB-SCs into the patient [9,14]. Furthermore, the trial confirms our expectation that co-culturing patient lymphocytes with CB-SCs alters the patient's immune response and leads to clinically relevant improvement in the autoimmune process. Previous studies that have demonstrated improved metabolic control in T1D have usually been limited to new- or recent-onset participants with residual β cell function [36-38], but this study demonstrates that Stem Cell Educator therapy is effective both in T1D with and without residual β cell function. Although we were not able to directly evaluate the status of islets or β cells through histological examination in this study, previous studies have demonstrated that patients with long-standing, severe T1D have lost all islets due to infiltration of autoimmune cells [39]. Thus, the successive improvement we observed in C-peptide levels (both fasting and OGTT) following Stem Cell Educator therapy suggests improvement in the number and/or function of islet β cells. The improvement of islet β cell function in T1D patients with residual islet β cells is impressive, but the recovery of islet β cell function in T1D patients without evident β cell function prior to treatment indicates Stem Cell Educator therapy addresses the underlying challenge of autoimmunity and controls the immune response sufficiently to allow regeneration of the native β cell population. Thus, this trial provides powerful evidence that exposing a patient's lymphocytes to CB-SCs can achieve the two essential outcomes required to cure T1D: reversal of autoimmunity and regeneration of islet β cells. However, longer post-treatment observations with larger samples are needed.

Importantly, the trial provides additional support for the mechanisms of CB-SC-mediated immune modulation and demonstrates that these mechanisms are apparent and lasting in patients. Specifically, the trial provides evidence that CB-SCs in the device educate effector T cells and/or Tregs, resulting in lasting changes in the expression of costimulating molecules, increasing the population of Tregs, and restoring Th1/Th2/Th3 cytokine balance, each of which is expected to improve control of autoimmunity of T1D [14,40]. Therapy also increases production of TGF-β1 in plasma of T1D subjects, one of the best-characterized cytokines contributing to the induction of peripheral immune tolerance [23]. Results from a NOD mouse study [14] demonstrated that increased plasma TGF-β1 may contribute to the formation of a 'TGF-β1 ring' around pancreatic islets that protects β cells against infiltrating lymphocytes, providing a safe environment for promotion of β cell regeneration [14,15]. Due to the limitation of obtaining pancreatic tissues from human subjects, evidence from our trial indicates that β cell regeneration occurs even in patients who do not appear to have β cells prior to treatment. CB-SCs from the device are not likely to be the source of this regeneration because they are not transferred to the patient during therapy. As demonstrated in other studies, the regenerated cells may be derived from multiple endogenous resources such duct cells, α cells [11,41], and peripheral blood-derived insulin-producing cells [42]. Further studies may provide additional insight into the role of TGF-β1 in β cell regeneration and the source of regenerated cells in T1D patients without functional β cell populations.

## Conclusions

In conclusion, findings from this study demonstrate the feasibility and safety of Stem Cell Educator therapy and demonstrate that T1D patients achieve improved metabolic control and reduced autoimmunity that lasts months following a single treatment. Further improvement may be achieved with additional treatments. Notably, our clinical data provide powerful evidence that reversal of autoimmunity leads to regeneration of islet β cells and improvement of metabolic control in long-standing T1D subjects. This principle may also be beneficial in the treatment of other autoimmune-related diseases.

## Abbreviations

Aire: autoimmune regulator; CB-SC: human cord blood-derived multipotent stem cells; HbA_1_C: glycated hemoglobin; HLA: human leukocyte antigen; ICOS; inducible costimulator; IL: interleukin; NOD: nondiabetic mouse; OGTT: oral glucose tolerance test; siRNA: small interfering RNA; SSEA: stage-specific embryonic antigen; TGF-β1: transforming growth factor-β1; Th: helper T cell; T1D: type 1 diabetes; Tregs: regulatory T cells.

## Competing interests

The authors declare that they have no competing interests.

## Authors' contributions

YZ, JZ, ZT, YM, HC, SR, PB, and TM designed the experiments. YZ, Yin Z, DY, and LY performed the clinical treatment and follow-up observations. LH and Ye Z prepared the stem cells. Ye Z, CY, SX performed laboratory experiments. FMB provided cord blood units. YZ, SR, HM, PB, and TM wrote the manuscript. All authors read and approved the final version of manuscript.

## Pre-publication history

The pre-publication history for this paper can be accessed here:

http://www.biomedcentral.com/1741-7015/10/3/prepub

## Supplementary Material

Additional file 1 **Additional file 1 on materials and methods**. 1. Supplemental methods. 2. Supplemental Figure S1. 3. Supplemental Figure S2. Click here for file
