# Supplementary material for: Reversal of type 1 diabetes via islet β cell regeneration following immune modulation by cord blood-derived multipotent stem cells
Source: BMC Med. 2012 Jan 10;10:3. doi: 10.1186/1741-7015-10-3 (PMC3322343; doi:10.1186/1741-7015-10-3)
Supplement: Additional file 1 — on materials and methods. 1. Supplemental methods. 2. Supplemental Figure S1. 3. Supplemental Figure S2. [file 1741-7015-10-3-S1.PDF]

Additional file 1:

Flow Analysis

Flow analysis was performed as previously described [1]. Cells were incubated with rat anti-human CD16 monoclonal antibody (eBioscience, San Diego, CA) diluted in medium containing 2.5% horse serum (Vector Laboratories) for 15 min at 4°C to block Fc receptors and to prevent non-specific staining. For cell surface staining, cells were incubated with rat anti-human monoclonal antibodies (eBioscience), including FITC-conjugated CD28, FITC-conjugated CD278 (ICOS), phycoerythrin (PE)-conjugated CD4, FITC-conjugated CD25. For intracellular cytokine staining, cells were initially stained for cell surface antigens (e.g., PE-conjugated CD4, and FITC-conjugated CD25) and then prepared by using a BD Cytofix/Cytoperm Fixation/Permeabilization kit (BD Biosciences, San Jose, CA). Subsequently, cells were stained with different combinations of antibodies including FITC-conjugated IL-4, PE-conjugated IL-5, PE-conjugated IL-12, FITC-conjugated IL-13, FITC-conjugated IL-17A (eBioscience), and Alexa Fluor 647-conjugated anti-Foxp3 (BD Biosciences). Cells were regularly stained for 45 min at 4°C and then washed with cold PBS prior to flow analysis. After staining, cells were analyzed using a Cytomics™ FC 500 (Beckman Coulter) or CyAn ADP (DakoCytomation). Isotype-matched rat anti-mouse IgG antibodies (eBioscience) served as negative control.

Western Blotting and siRNA Knockdown

Western blotting was performed as previously described [2]. Cells cultured in Petri dishes were washed with PBS and then solubilized with RIPA buffer (150 mM NaCl, 0.5% sodium deoxycholate, 0.1% SDS, 1% Triton X 100, 20 mM Tris (PH 8.0), 5 mM EDTA) with a cocktail of protease inhibitors (Sigma). Proteins samples (20 µg each) were mixed with a loading buffer (50 mM Tris, 10% SDS, 10% glycerol, 10% 2-

mercaptoethanol, 2 mg of bromphenol blue) in a volume ratio of 1:1, boiled, loaded, and separated by electrophoresis on 10% SDS gels. The separated proteins were then transferred to a nitrocellulose membrane, blocked with 5% non-fat dry milk in TBST for 1h and incubated with different antibodies: including rabbit anti human Aire Ab (1:500) and mouse anti-human PD-L1 Ab (1:1,000), diluted in PBST for 1h at room temperature. After washing, the blot was exposed to a horseradish peroxidase-conjugated secondary antibody (1:2,000; Pierce) in PBS-T. The immunocomplexes were visualized by the enhanced chemiluminescence (ECL, GE healthcare) method. β-actin served as an internal loading control. To further confirm that Aire signaling plays a critical role in the modulation of CB-SC, the small interfering RNAs (siRNAs) for the transcription factor Aire were used in cell cultures using Lipofectamine RNA iMAX transfection as previously described [3].

Immunocytochemical Analysis

Immunocytochemical evaluation was performed with the use of a commercial kit, as described previously with minor modifications [1,4]. To block non-specific staining, sections were incubated in a buffer containing 2.5% horse serum (Vector Laboratories) for 20 min at room temperature. Rabbit anti-Aire Ab was purchased from Abcam, Cambridge, MA. After incubation with primary antibody, cells were stained with an ABC kit (Vector Laboratories, Burlingame, CA). Biotinylated horse anti-rabbit Ab and biotinylated goat anti-guinea Ab were purchased from Vector Laboratories (Burlingame, CA). For isotype-matched controls, rabbit IgG was purchased from BD Biosciences. For every experiment, isotype-matched antibody was used as negative controls. Cells were photographed with a Zeiss Axiocam Color Camera using Zeiss Axioskop Histology/Digital Fluorescence microscope for HRP-immunostaining images.

Cytokine Assays

Cytokine levels in human plasma were quantified using commercial ELISA kits following manufacturer's instructions. We purchased human IL-10 and TGF-β1 ELISA kits from Biolegend Inc.(San Diego, CA).

Oral Glucose Tolerance Testing (OGTT)

Subjects were fasted overnight (12 h) and did not drink anything except water until the test was completed. Also, subjects minimized their activity such as walking or exercise in the day before and the morning of the test. Before the OGTT test, a heparin lock was inserted into a vein of the patient's arm. A blood sample was then taken as baseline. After this, subjects were given a glass of liquid to drink, containing 75 g glucose. After drinking the liquid, samples of blood were taken at regular intervals (30, 60, 90, and 120 min) to measure glucose, insulin, and C-peptide levels.

References

1. Zhao Y, Lin B, Darflinger R, Zhang Y, Holterman MJ, Skidgel RA: **Human cord blood stem cell-modulated regulatory T lymphocytes reverse the autoimmune-caused type 1 diabetes in nonobese diabetic (NOD) mice.** *PLoS ONE* 2009, **4:** e4226.

2. Zhao Y, Huang Z, Lazzarini P, Wang Y, Di A, Chen M: **A unique human blood-derived cell population displays high potential for producing insulin.** *Biochem Biophys Res Commun* 2007, **360:** 205-211.

3. Zhang X, Tan F, Zhang Y, Skidgel RA: **Carboxypeptidase M and kinin B1 receptors interact to facilitate efficient b1 signaling from B2 agonists.** *J Biol Chem* 2008, **283:** 7994-8004.

4. Zhao Y, Wang H, Mazzone T: **Identification of stem cells from human umbilical cord blood with embryonic and hematopoietic characteristics.** *Exp Cell Res* 2006, **312:** 2454-2464.

Figure legends for supplementary figures:

Figure S1. Flow analysis with the CB-SC-specific marker stage-specific embryonic antigen (SSEA)-3 failed to show CB-SC leaving the Educator and transferred to the patient. After treatment with the Stem Cell Educator, cells returned to patients were collected for flow cytometry. Leukocyte common antigen CD45 served as positive control (right panel, red line). Isotype-matched IgG served as negative control (grey line).

Figure S2. Markers of immune function in control T1D patients at baseline and 4 weeks after Stem Cell Educator therapy. Flow analysis of intra-cellular cytokines demonstrating no differential effects on key interleukins at 4 weeks post treatment.

Figure S1

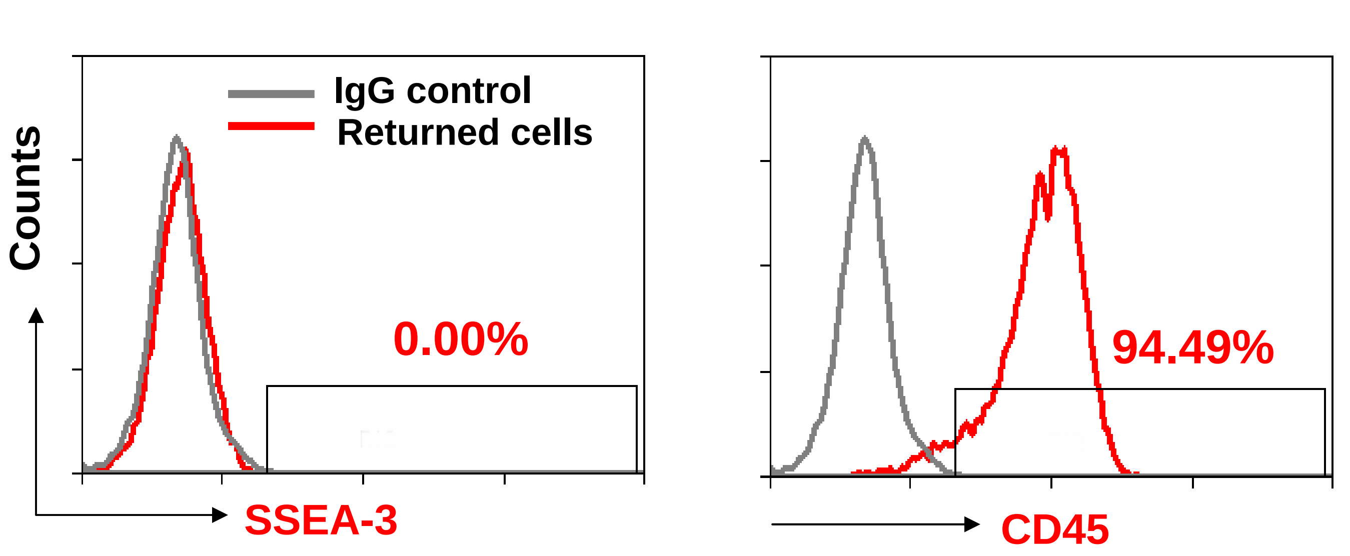

Figure S2

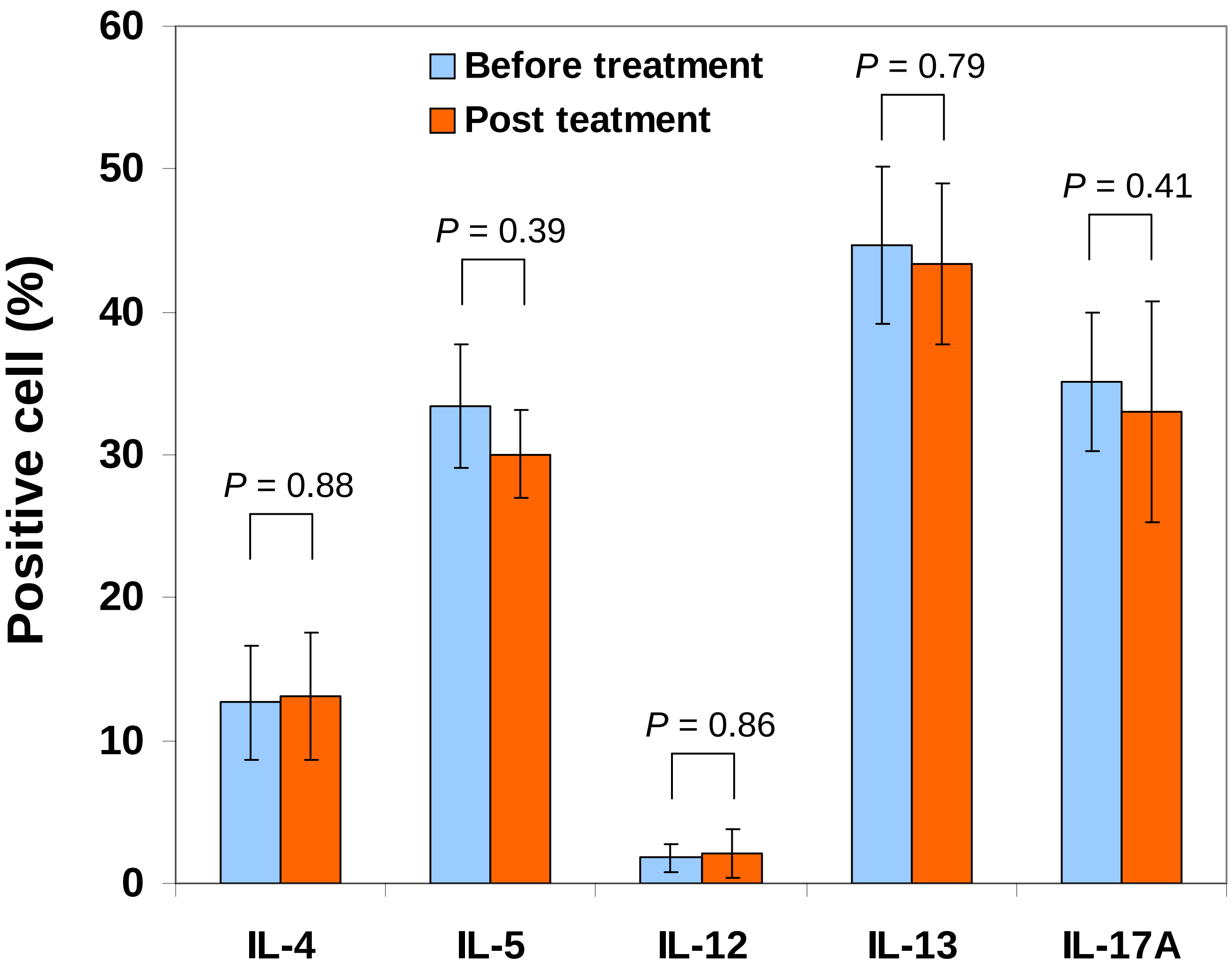

Positive cell (%)
60
50
40
30
20
10
0
Before treatment
Post teatment
P = 0.88
P = 0.39
P = 0.86
P = 0.79
P = 0.41
IL-4
IL-5
IL-12
IL-13
IL-17A
